# Optimization of catalytic wet oxidating fulvic acid with zero-valent copper chitosan activated carbon ball as the catalyst

**DOI:** 10.1038/s41598-021-92789-6

**Published:** 2021-07-07

**Authors:** Chaofei Song, Yue Lv, Xia Qin, Chengrui Guo, Jiaxin Cui, Wendkuuni Steve-Harold Kaghembega

**Affiliations:** Faculty of Environment and Life, Beijing University of Technology, Beijing, 100124 China

**Keywords:** Pollution remediation, Environmental sciences, Catalyst synthesis

## Abstract

The degradation efficiency of fulvic acid (FA) was investigated in the catalytic wet oxidation process (CWPO) by zero-valent copper chitosan activated carbon ball (ZVC/CTS-ACB). Characterization of ZVC/CTS-ACB shows that zero-valent copper was loaded successfully on the chitosan activated carbon. Plackett–Buiman (PB) design and response surface methodology (RSM) were employed to determine the influence factors and the optimum processing parameters. The model was well fitted to the actual data and the correlation coefficients of R^2^ and R^2^-adj were 0.9359 and 0.9039, respectively. Under the obtained optimum conditions for FA degradation: temperature = 94 °C and pH 3.8, the average FA removal by three replicate experiments was 93.02%, which has a high consistency to the RSM optimal target response of 93.86%. The comparison of catalytic performance showed that the addition of catalyst ZVC/CTS-ACS could increase the removal rate of FA, color number (CN) and TOC by 93.6%, 83.5% and 81.9% respectively. The high TOC removal rate indicated the good performance of the catalyst to FA mineralization. Additionally, the ICP analysis of copper ion leaching was only 0.08 mg/l after 5 repeated recycles of the catalyst, demonstrating the high stability of ZVC/CTS-ACB that is beneficial for the actual application.

## Introduction

Fulvic acid (FA), which is one major component of humic substances (HSs)^1–3^, can be found in both natural waters and wastewater, especially in landfill leachate^1^. In the broadest terms, the structures of HSs can be described as assemblies of covalently linked aromatic and aliphatic residues carrying carboxyl, phenolic and alkoxy groups^2^. These common functional groups of FA make it possible that FA cause many serious environmental and health problems^3^. FA has strong complexation ability with heavy metals, leading to the formation of organometallic complexes with increased transportation ability, bioavailability and toxicity^4^. In the chlorination stage of the water treatment, FA and chlorine may produce a by-product trihalomethane whose strong carcinogenic and mutagenic properties seriously endanger human health^5–7^. Due to the complex structure, FA is frequently identified as refractory organic matter^8^. Therefore, biological methods are ineffective for FA treatment and many physical, chemical and physico-chemical methods have been employed for FA removal^9^. The advantages and disadvantages of these methods are summarized in Table 1.

**Table 1:** The methods of FA removal.

	Advantages	Disadvantages
Coagulation and flocculation	Removing macromolecular colloidal organics effectively; low operation cost	Requires using more chemicals, leading to the generation of more sludge
Adsorption	Convenience, ease of operation, and simplicity of design; removing organic matter with molecular weight of 500–3000 da effectively	Macromolecular organic compounds are easy to block the pore structure of adsorbent; not suitable for high concentration wastewater
Membrane filtration	Removing organic matter, salt and pathogenic microorganisms in water effectively	Causing irreversible membrane fouling by pore narrowing and the formation of loose cake layer; the membrane concentrate should be treated further
Advanced oxidation processes	Removing all kinds of organic matter effectively without selectivity	High operating costs

Catalytic Wet Hydrogen Peroxide Oxidation (CWPO) is a kind of Advanced Oxidation Processes (AOP)^10–13^. The efficiency of CWPO for the degradative removal of organic matters from wastewater is mainly based on high amounts of hydroxyl radicals (·OH) from the reaction of catalyst and H_2_O_2_. The generated hydroxyl radicals are highly reactive species and thus can unselectively react with organic matters and convert them into smaller molecular products, and even directly into CO_2_ and H_2_O^14,15^. The advantages of high efficiency and low intermediate pollutants make the CWPO process more attractive for treating complex organic wastewater^16,17^. The degradation efficiency of CWPO can be improved by enhancing the catalyst activity. Zero valent metals including nanoscale zero valent iron (nZVI)^18,19^, zero valent aluminum (ZVAl)^20^, and zero valent copper (ZVC)^12,21^, could activate molecular oxygen to induce the generation of more powerful reactive oxygen species (ROS), such as superoxide radical anion (·O_2_^−^) and hydroxyl radical (·OH). Copper is one of the major redox-active transition metal catalysts, but the application of Cu catalyst in CWPO was severely limited due to the instability of Cu^+^. However, ZVC has a potential capacity to release Cu^+^ through oxidation to activate H_2_O_2_ to generate ·OH thereby resulting in the degradation of organic contaminants^12,20^.

Activated carbons have good performance in adsorption and catalysis^22^. Activated carbons can be produced through either physical or chemical activation. Physical activation involves pyrolysis of a carbonaceous precursor to produce a char follower by some activating agents such as carbon dioxide or steam. In the chemical activation, the precursor was pretreated with chemical activator (e.g. ZnCl_2_, H_3_PO_4_, KOH and NaOH), and then thermal treated via pyrolysis to produce an activated carbon^23^. Williams^24^ produced activated carbon via both physical and chemical activation with biomass waste flax fibre as precursor. The results showed that physical activation produced activated carbons with a nodular and pitted surface morphology whereas activated carbons produced through chemical activation had a smooth surface morphology. TEM analysis could identify mesoporous structures in the physically activated carbon and microporous structures in the chemically activated carbons. Sahira et al.^25^ used different activating agents (KOH, H_2_SO_4_, FeCl_3_, MgCl_2_ and CaCl_2_), and found out that the different activating agents have no significant effect on the nature of surface functional groups, with all showing similar oxygenated functional groups in FT-IR such as hydroxyl, carbonyl, carboxyl and lactones. Ahmed et al.^26,27^ prepared activated carbon via two-stage activation using H_3_PO_4_ and KOH and the produced activated carbon possessed high surface area (692.3 and 1368 m^2^/g) and pore volume (0.44 and 0.92 cm^3^/g).

Chitosan (CS), the second most abundant biopolymer after cellulose in nature and derived from chitin of crab shells and fungus cell walls^28^, has gradually attracted the attention of researchers^28–32^ due to its non-toxicity and biocompatible, cost-effective nitrogen precursor, and environmentally friendly features. Hitherto, CS and its derivatives were used in different areas including flocculating agents, adsorbents, catalyst, thickeners, food preservation, and many others^33,34^. Recently, a variety of carbon materials has been developed from CS and used in different applications, thereby highlighting its great impact on chemistry and material science^35^. He et al. prepared activated carbon by surface modification with CS as nitrogen source during KOH activation process, displaying a remarkable CO_2_ uptake achievement of 0.00583 mol/g^36^. In addition, CS contains abundant hydroxy and amine functional groups, which are responsible for its easy incorporation with the other materials^31^. CS can serve as a chelating agent for 3d metal ions such as Fe^3+^, Cu^2+^ and Ni^2+^ because of its flexible structure and high chemical reactivity^32^. The CS-derived metal–carbon nanocomposites can serve as catalysts due to their textural properties, the presence of nitrogen species, and the uniform dispersion of metal nanoparticles^35^. Guo et al.^37^ prepared the chitosan-supported iron (III) tetraphenylporphyrin. In their study, under reaction conditions of 418 K and 0.8 MPa, the cyclohexane oxidation catalyzed by chitosan-supported iron (III) tetraphenylporphyrin had 10.48% cyclohexane conversion and 79.20% cyclohexanone and cyclohexanol selectivity. Wang et al.^38^ prepared the Cu–M@CS–SiO_2_ degrading 1,1-dimethyl hydrazine (UDMH) wastewater. The COD removal of UDMH wastewater was 87.38% in half an hour.

Traditionally, the CWPO experimental conditions have been optimized by several single-factor or orthogonal experiments, in which the optimal conditions can be found only within the chosen experiment points. Additionally, these traditional methods also ignore the interaction among some parameters^37^. Plackett–Burman (PB)^39,40^ design is a statistical method for screening the significant influence factors in multi-factors experiments. The statistical experiment design of the response surface method (RSM) can optimize the values of all the influence parameters including the interaction factors and provide an optimal prediction model for the experiments. RSM can even find the optimal target response point outside the set condition interval^41^.

In this study, chitosan was used to produce stable chelates with copper ions and then the chitosan chelates was carbonated to provide well-distributed ZVC active sites^42^. The chitosan-activated carbon was used as both an adsorbent for adsorbing organic compounds to the reaction site and more importantly a carrier to support active metals of the catalyst. Additionally, carbon was used to reduce Cu^2+^ to Cu^0^ during the pyrolysis, which makes the catalyst perform better in the CWPO. The purpose of this study was to evaluate the efficiency of FA removal in the CWPO process with ZVC/CTS-ACB as catalyst. The RSM coupled with PB was used to optimize the parameters of the CWPO experiment. The catalyst was prepared and the catalytic performance in CWPO was investigated in comparison with other degradation processes in removal of FA, TOC and colour number (CN).

## Materials and methods

### Materials

FA was purchased from Cool Chemical Technology Corporation (Beijing). The appearance color of the FA solution varies from dark yellow to light yellow depending on the concentration. Chitosan was supplied by Jinan Haidebei Marine Bioengineering, whose Deacetylation Degree ≥ 85%, Particle ≥ 40 Mesh and viscosity = 200 mpas. All other agents were of analytical grade and purchased from Beijing Chemical Plant. The pH of the FA solution was adjusted by adding H_2_SO_4_ or NaOH. All the water used in the experiment was deionized water, from a Millipore-Q system with a resistance of 18.2 MΩ.

### Preparation and characteristics of ZVC/CTS-ACB

The preparation of ZVC/CTS-ACB was carried out according to the following steps: (1) Preparation of chitosan gel: 7.25 g of CuNO_4_·3H_2_O was weighed and dissolved in 960 ml of deionized water, at the same time 0.1 g of citric acid was added, and then 30 g of chitosan was weighed and dispersed in a copper nitrate solution to form a suspension. 40 ml of acetic acid was added and stirred quickly and uniformly until a gel was formed. The gel was allowed to stand overnight to discharge air bubbles. (2) The gel was added dropwise to a 3.75 to 5 wt% sodium hydroxide solution using a syringe, then allowed to stand for 4–6 h. (3) After washing to neutral and drying at 60–100 °C for one night, the precursor was kept at 800–850 °C for 2–5 h in an inert gas tube furnace to complete its carbonization and become an activated carbon ball. (4) The activated carbon ball was rinsed several times in ethanol and deionized water, and then dried at 80 °C to remove moisture.

### Experimental studies

The CWPO reaction was carried out in a reactor whose temperature can be controlled. The pH value of the water sample was adjusted by 0.5 M H_2_SO_4_ and 1 M NaOH solutions, and an appropriate amount of ZVC/CTS-ACB was added to the water sample. When the reaction temperature reached the set temperature, 30% of H_2_O_2_ was dropped into the reactor through the transfer pipe to make hydrogen oxide fully contact the catalyst to produce strong oxidizing free radicals^43^, such as ·OH, ·O_2_, etc. These free radicals can oxidize organics into small molecular organics and even H_2_O and CO_2_. Samples were taken every ten minutes from the reaction effluent under different conditions. MnO_2_ was added to the sample to prevent the influence of excess H_2_O_2_ on the detection results, and then the sample was filtered through a 0.45 μm membrane for the subsequent results testing.

### Analytical methods

The surface and profile morphology of the catalyst and the distribution of the supported metal crystals were observed using SEM (FEI Quanta 200). The active crystal phase composition of the catalyst was identified by XRD (D8 Advance type). The element composition was analyzed by the XPS. The chemical group was detected by the FT-IR (IRPrestige-21). Total copper ion in solution was quantified by the ICP. The pH value was measured using a pH meter (pHs-3C type). The total organic carbon (TOC) of the influent and effluent was analyzed using a total organic carbon analyzer (TOC-5000A, Shimadzu, Japan), and the mineralization rate (α, %) was calculated by Eq. (1).

1 $$\upalpha = \frac{{{\text{TOC}}_{0} - {\text{TOC}}}}{{{\text{TOC}}_{0} }} \times 100\% ,$$

where TOC_0_ is the initial concentration and TOC is the effluent concentration.

The absorbance of FA was measured at a wavelength of 254 nm using an Ultraviolet–Visible spectrophotometer (Hitachi U-3900). The removal rate (β, %) of the FA is calculated by Eq. (2).

2 $${{\upbeta }} = \frac{{{\text{UV}}_{{254\left( 0 \right)}} - {\text{UV}}_{{254}} }}{{{\text{UV}}_{{254\left( 0 \right)}} }} \times 100\% .$$

The color number of FA can be calculated and analyzed by UV–Vis^44^. Since the visible region of the leachate spectrum showed no limited absorption maxima, the colour number (CN) defined by Eq. (3) was used to characterize the colour. CN relies on the measurement of the spectral absorption coefficient (SAC) in the visible range at wavelengths of 436, 525 and 620 nm. SAC is determined by the absorption value (Abs) divided by a cell of thickness x, which is shown in Eq. (4).

3 $$CN = \frac{{SAC_{{436}}^{2} + SAC_{{525}}^{2} + SAC_{{620}}^{2} }}{{SAC_{{436}} + SAC_{{525}} + SAC_{{620}} }},$$

4 $$SAC_{i} = \frac{{Abs_{i} }}{x}.$$

The CN removal rate (γ, %) of FA is calculated by Eq. (5).

5 $$\gamma = \frac{{CN_{i} - CN_{0} }}{{CN_{i} }} \times 100\% .$$

### Plackett–Burman and response surface methodology

The Design Expert Software (version 8.0) was used for experiment design and data analysis. Plackett–Burman (PB) coupled with central composite design response surface methodology (CCD-RSM) were used to evaluate and optimize the impact factors. Taking the removal rate of FA as the response target, two steps were used to optimize the experimental factors: Firstly, the PB experimental design was used to select the factors that significantly influenced the response, then CCD of RSM is used to find the optimum experimental conditions. During the experiment period, the CWPO was performed to degrade FA according to the designed conditions, the absorbance values of the FA samples before and after the reaction were measured at a wavelength of 254 nm, and the removal rate of the target fulvic acid was calculated by Eq. (2). After the experiment, the second-order response surface model equation was obtained and the optimal experimental conditions were determined by PB and CCD-RSM, and finally the optimal conditions were verified by conducting three repetitive tests.

### Plackett–Burman experiment

The Plackett–Burman experimental design was proposed by Plackett and Burman in 1946. It was based on the principle of incompletely balanced plates. At most (N − 1) variables (N was generally a multiple of 4) could be studied by N experiments^45^. During the experiment, dummy variables are usually reserved as error analysis. Each variable has two levels, high and low, marked as (+) and (−), respectively.

The Plackett–Burman design of the CWPO experiment was shown in Table 2. The seven main factors of the FA degradation experiment were screened, plus four dummy variables. Each variable was determined at two levels (+) and (−), and a total of 12 experiments were conducted to determine the impact factors.

**Table 2:** Factors of PB design experiment.

Variable	X1:A	X2:B	X3:C	X4:D	X5:E	X6:F	X7:G	X8, X9, X10, X11 (H, I, J, K)
Factors	Temperature/°C	Initial volume/ml	Initial FA concentration/mg/L	Time/min	H_2_O_2_/mmol	(ZVC/CTS-ACB)/g/L	Acidity	–
High level (+)	90	250	100	45	10	3	4	–
Low level (−)	60	500	200	90	20	5	7	–

### Response surface methodology

The Central Composite Design (CCD) developed by Box and Wilson is a commonly used response surface design method, with which an optimal fitted model can be obtained with minimum numbers of experiments. The second-order empirical model is generally used to characterize the response behavior of variables.

$$Y = \beta _{0} + \sum\limits_{{i = 1}}^{k} {\beta _{{\text{i}}} X_{i} } + \sum\limits_{{i = 1}}^{{j - 1}} {\sum\limits_{{j = 1}}^{k} {\beta _{{{\text{ij}}}} } } X_{i} X_{j} + \sum\limits_{{i = 1}}^{k} {\beta _{{{\text{ii}}}} } X_{i}^{2} ,$$

where: Y represents the system response; β_0_, β_i_, β_ii_ are the offset term, linear offset and second-order offset coefficient, respectively; β_ij_ is the interaction coefficient; X_i_ is the horizontal value of each factor.

### Comparative experiment of catalytic performance

In order to better understand the role of the catalyst (ZVC/CTS-ACB) in the CWPO process, the UV_254_, TOC and CN removal efficiency of FA was investigated in various CWPO systems.

## Results and discussion

### Characterization of ZVC/CTS-ACB

**Figure 1:**
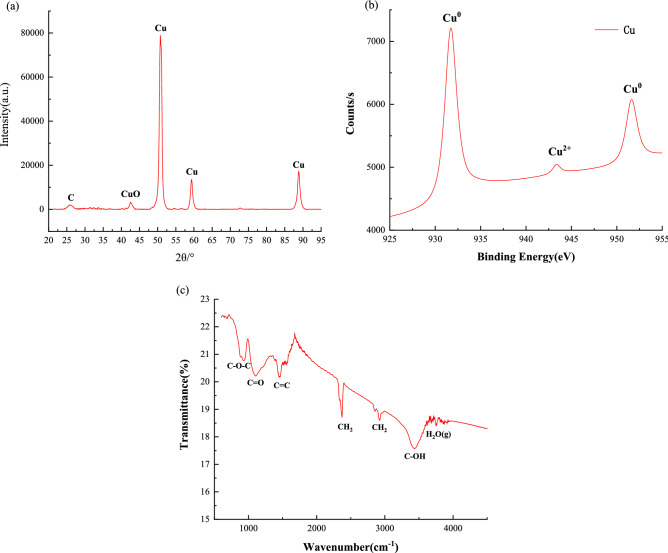
(**a**) XRD pattern of ZVC/CTS-ACB. (**b**) XPS of ZVC/CTS-ACB. (**c**) FT-IR of ZVC/CTS-ACB.

**Figure 2:**
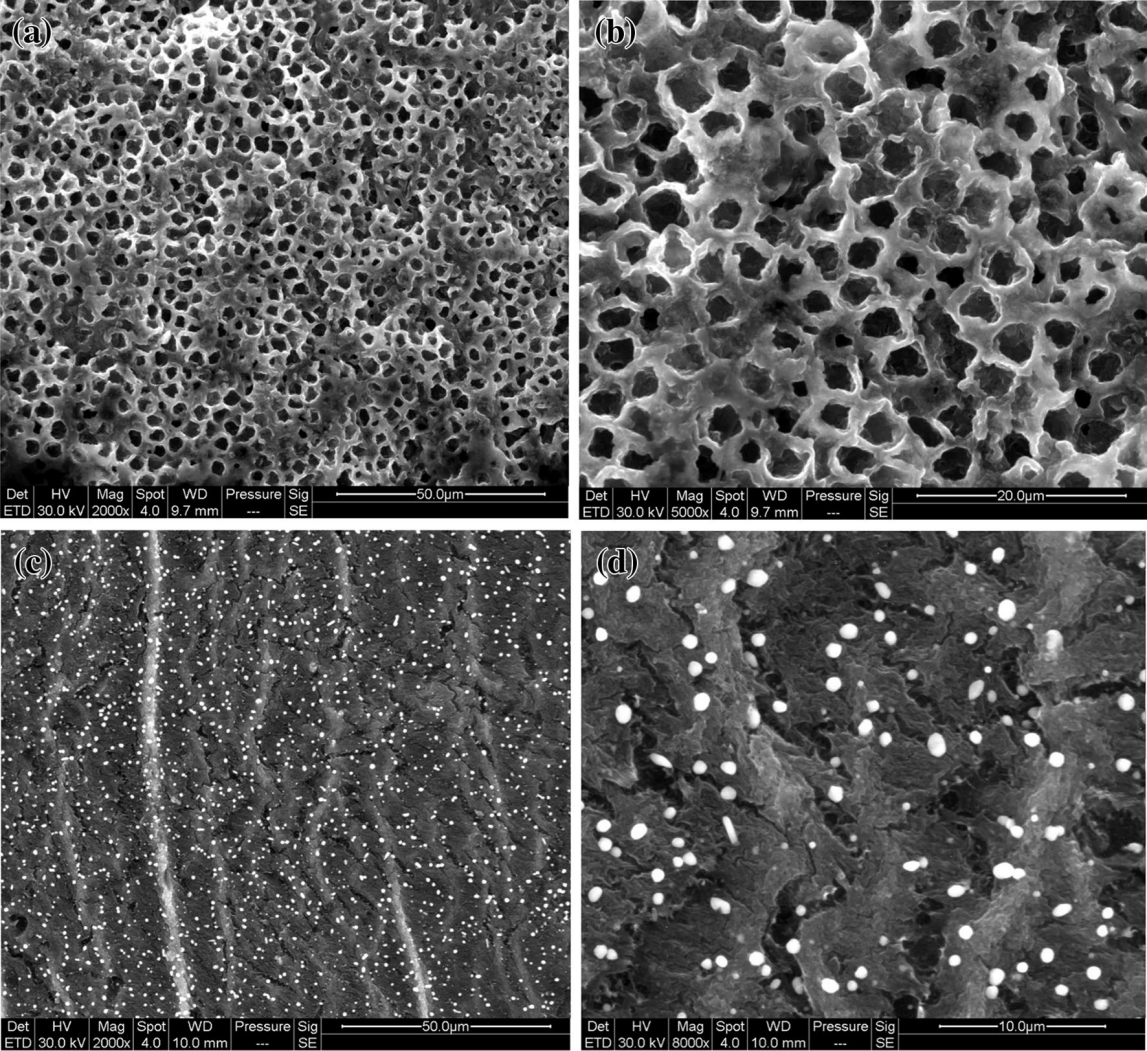
(**a**) SEM of outer surface is magnified 2000 times of ZVC/CTS-ACB. (**b**) SEM of outer surface is magnified 5000 times of ZVC/CTS-ACB. (**c**) SEM of inner cut surface is magnified 2000 times of ZVC/CTS-ACB. (**d**) SEM of inner cut surface is magnified 8000 times of ZVC/CTS-ACB.

The composition of the ZVC/CTS-ACB was characterized by X-ray diffraction (XRD) and X-ray photoelectron spectrometry (XPS). The diffraction peaks at 43.46°, 50.56° and 74.31° match well with the standard pattern of zero-valent copper (JCPDS 85-1326) (Fig. 1a)^46^. The crystalline grain size of Cu was calculated as 7.76 nm by the Williamson–Hall method. The Cu2p XPS spectrum (Fig. 1b) showed the peak of CuO at 943.3 eV as well as peaks of Cu^0^ at 931.7 eV and 951.6 eV^46^. All results showed that zero-valent copper existed in the catalyst. The absorption bands for ZVC/CTS-ACB (Fig. 1c) are 925.8, 1099.4 and 3437.1 cm^−1^, which correspond to C–O–C stretching, C=O bending and O–H stretching. All of these indicate the carboxyl groups on ZVC/CTS-ACB^47^, which may facilitate electron transfer^48^. Furthermore, the scan electron microscopy (SEM) morphologies (Fig. 2) showed that the ZVC/CTS-ACB have porous network structure on the surface, and the cross-sectional view showed uniform distribution of copper microcrystalline particles in the interior. The BET results presented that the specific surface area, pore volume and pore size were 42.4 m^2^/g, 0.98 cm^3^/g and 1.9 nm, respectively.

### Plackett–Burman experimental design results and analysis

The results of 12 runs of the FA removal experiments are shown in Table 3. The analysis of variance (ANOVA) results are shown in Table 4 with a list of significant differences of each factor impact.

**Table 3:** PB design and response values.

Run	Factor: A	Factor: B	Factor: C	Factor: D	Factor: E	Factor: F	Factor: G	Response (%)
1	−	+	−	−	+	−	+	37.77
2	+	+	−	+	+	+	−	84.59
3	+	−	+	+	−	−	+	51.13
4	+	+	−	−	+	−	+	48.41
5	+	−	−	−	−	+	+	45.59
6	−	+	+	+	−	+	+	34.38
7	−	−	+	−	+	+	−	74.54
8	+	−	+	+	+	−	−	83.11
9	−	−	−	+	+	+	+	41.07
10	−	−	−	−	−	−	−	73.32
11	−	+	−	+	−	−	−	78.29
12	+	+	+	−	−	+	−	81.26

**Table 4:** ANOVA results.

Source	Mean square	F value	Value	Remarks
Model	525.97	51.46	0.0040	Significant
A	197.94	19.37	0.0218	Significant
B	1.66	0.16	0.7141	Not significant
C	6.21	0.61	0.4926	Not significant
D	8.32	0.81	0.4357	Not significant
E	2.18	0.21	0.6753	Not significant
F	8.32	0.81	0.4335	Not significant
G	338.98	37.13	0.0041	Significant

As shown in Table 4, the Model’s F-value of 51.46 proves the model is significant. There is only a very small chance (< 0.004) that the F-value of the model is the result of noise. In this case, A, G are significant model terms. Values greater than 0.1000 indicates the model terms are not significant. It could be concluded that only the temperature and acidity factors have significant effects on the target values among the seven factors considered. It has been reported that the higher the reaction temperature, the faster the reaction^49^, and that pH can affect the chemical reaction rate and the production of free radicals^50^. Therefore, temperature and acidity were selected for the following central combination design.

### CCD optimization design results and response surface analysis

The CCD was conducted for the Temperature and acidity selected by Plackett-Buiman with other non-critical factors fixed: Initial volume = 250 ml, Initial concentration = 100 mg/l, Time = 60 min, H_2_O_2_ = 20 mmol, (ZVC/CTS-ACB) = 3 g/l. The CWPO degradation FA experiment was performed according to the designed conditions, and the target response value was calculated.

### Model fitting and analysis of variance

Table 5 shows the conditions of 13 runs and the value of FA removal rates. Evaluation of data in this table provides a second-order polynomial to express the relationship between FA removal efficiency and the experimental parameters.

$$Y = 80.25 + 15.98A + 20.28B - 6.64A^{2} - 25.70B^{2} ,$$

where: Y is the target response value, i.e. the removal of the FA. A and B represent temperature and acidity, respectively.

**Table 5:** CCD experimental design table.

Run	1	2	3	4	5	6	7	8	9	10	11	12	13
Factor A: temperature (°C)	90	90	70	50	70	70	98	42	70	70	70	70	50
Factor B: acidity	5.0	1.0	3.0	1.4	1.2	5.8	1.2	5.8	3.0	3.0	3.0	3.0	5.0
Response: removal rate of FA (%)	80.78	44.03	84.78	34.89	30.44	62.99	91.80	41.45	80.56	74.94	84.78	80.80	46.60

Table 6 shows the results of the RSM model fitting in the form of an analysis of variance (ANOVA). According to the table, the high F-value (F-value = 29.21) and the very low probability values (P value < 0.0001) indicates the model obtained is highly significant. At the same time, the F-value of the missing term is 4.66, indicating that the missing term has no significant effect, so the established model can be referenced^51^. From the corresponding P-value, it was shown that among the tested variables, the B-acidity and A-temperature value had the greatest influence on the removal efficiency of FA, and the surface effect of factor B^2^ on the FA removal effect is significant.

**Table 6:** ANOVA for the regression quadratic model of CCD design.

Source	Sum of squares	Df	Mean square	F value	P-value	
Model	5405.22	4	1351.31	29.21	< 0.0001	Significant
A-temperature	2029.59	1	2029.59	43.87	0.0002	
B-acidity	2208.68	1	2208.68	47.74	0.0001	
A^2^	311.26	1	311.26	6.73	0.0319	
B^2^	2679.40	1	2679.40	57.92	< 0.0001	
Residual	370.11	8	46.26			
Lack of fit	304.72	4	76.18	4.66	0.0826	Not significant
R-squared	0.9359					
Adj R-squared	0.9039					

The correlation system R^2^ is an important reference for the degree of fit. When R^2^ tends to be unified, the fit of the empirical model of the actual data is better. Joglekar and Ma^52^ suggested that for a good fit of the model, R^2^ should be at least 0.80. The R^2^ value of this model was 0.9359, indicating that the regression model fits well with the experiment.

### RSM analysis

**Figure 3:**
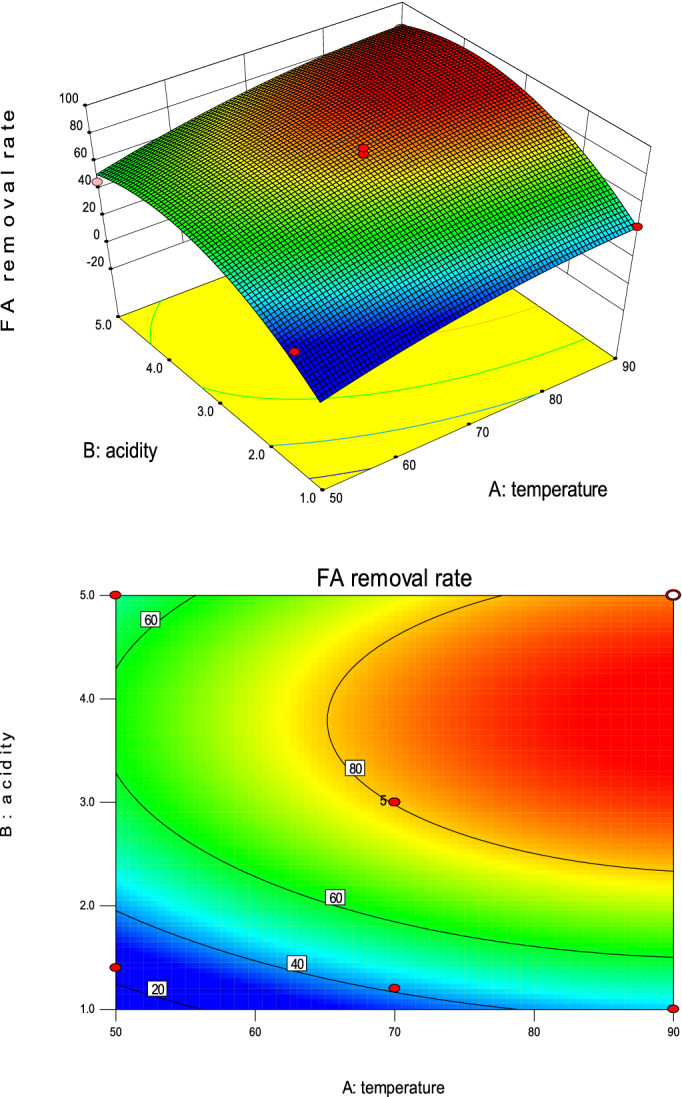
3D response surface graph and contour plot.

Figure 3 is a response surface graph and its contour plot from the multiple regression Eq. (3). It can be seen that the 3D response surface graph presents an inverted “U” shape, and a “red peak” appears at the center of the right side, meaning that the target optimal response value is obtained near here^53^. In the case of a fixed temperature, the target response value first increases and then tends to be gradual, and finally decreases slowly. On the other hand, if the acidity value is fixed, the target response value increases slowly and slightly with the increasing temperature. This also demonstrated that the degree of influence of acidity on the target FA removal was greater than temperature. The shape of the contour plot indicates the nature and extent of the interaction. The regular elliptical nature of the contour plot shows significant interactions, while the near-circular nature of the contour plots shows less prominent or negligible interactions^53^. It can be seen from the contour plot in Fig. 3 that there is no strong interaction between the influencing factors A and B here, which is consistent with the results obtained by the ANOVA above (Table 6). From the contour plot, the optimal acid of the model could be obtained in the acid pH range of 3 to 5, whereas the optimal temperature could not be found, it might be over 90 °C, so the model did need further optimization.

### Optimization of influencing factors

**Figure 4:**
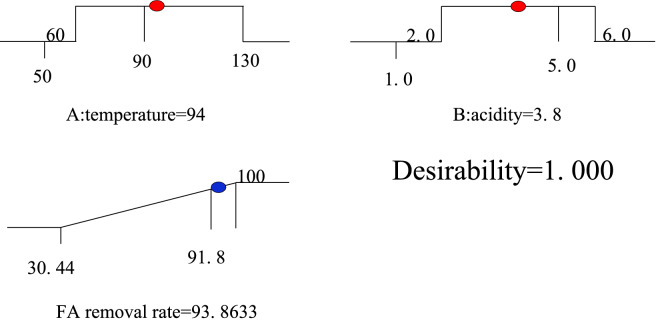
Desirable slope for numerical optimization of the CWPO conditions.

The main purpose of the optimization was to determine the optimal parameter values for maximizing the FA removal. Therefore, the maximum removal of FA was selected as the target value. Since the optimal temperature may emerge over 90 °C, the temperature range was enlarged from 60–90 to 60–130 °C. Then the optimum values were obtained, as shown in Fig. 4, whereby two optimization schemes were also obtained in Table 7. Since the conditions of the two schemes were very close, and the expected target values are not different from each other, the temperature and acidity were finally selected as 94 °C and pH 3.8 respectively for the convenience of the experimental conditions setting.

**Table 7:** RSM system optimized solution.

Solution	Temperature	Acidity	FA removal rate (%)	Desirability
1	94.07	3.8	93.8643	1.000
2	93.99	3.8	93.8622	1.000
Prediction	94	3.8	93.8633	1.000

**Figure 5:**
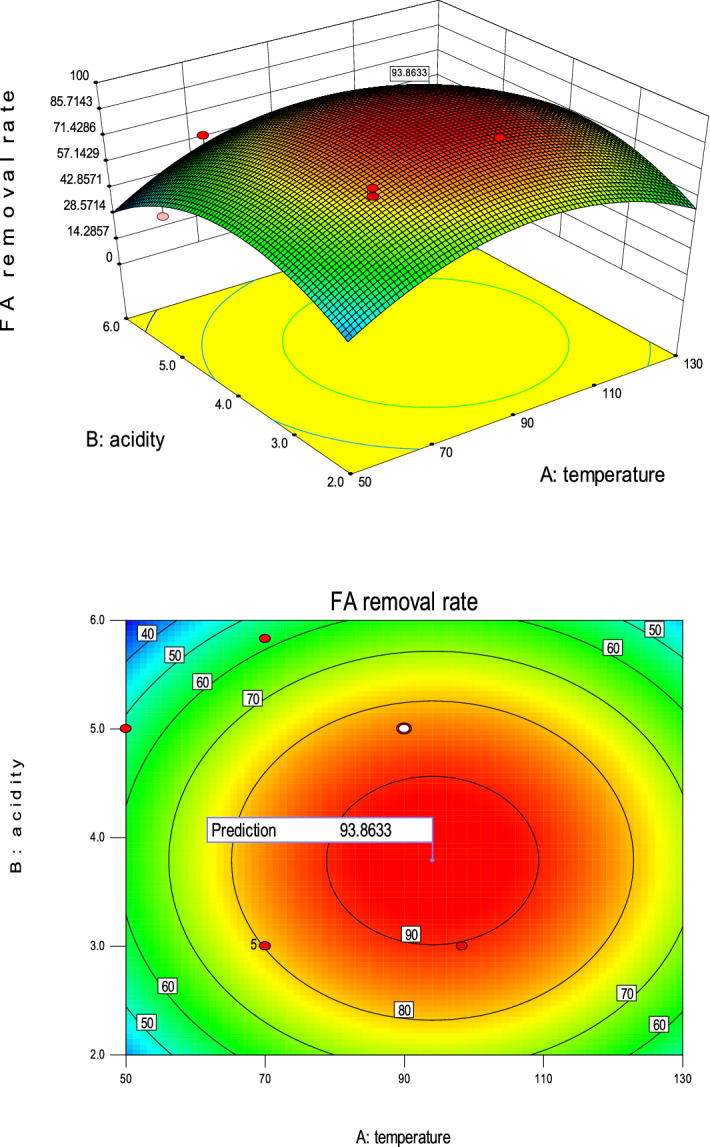
Optimized 3D surface graph and contour plots.

Figure 5 is the optimized 3D surface graph and contour plots. Compared with Fig. 3, a mountain shape 3D surface graph was obtained and it was obvious that the maximum removal of FA (93.8633%) was at the peak of the mountain.

### Results verification

To confirm whether the model is sufficient to predict the maximum FA removal efficiency, three repetitive tests were performed under the optimal operating condition as shown in Table 8. The average maximum FA removal efficiency produced by three replicate experiments was 93.02%, while the RSM optimal target response was 93.86%. The good agreement between the predicted results and the experimental results verifies the validity of the model and confirms that CCD-RSM is a powerful tool for optimizing the experimental factors.

**Table 8:** Optimum value of the process parameter for maximum efficiency.

Parameter	Y (removal efficiency, %)		
**Practice**			
Times	1	2	3
Single	92.45	92.82	93.78
Average	93.02		
Prediction	93.86		

### Removal efficiency under different systems and the stability of ZVC/CTS-ACB

**Figure 6:**
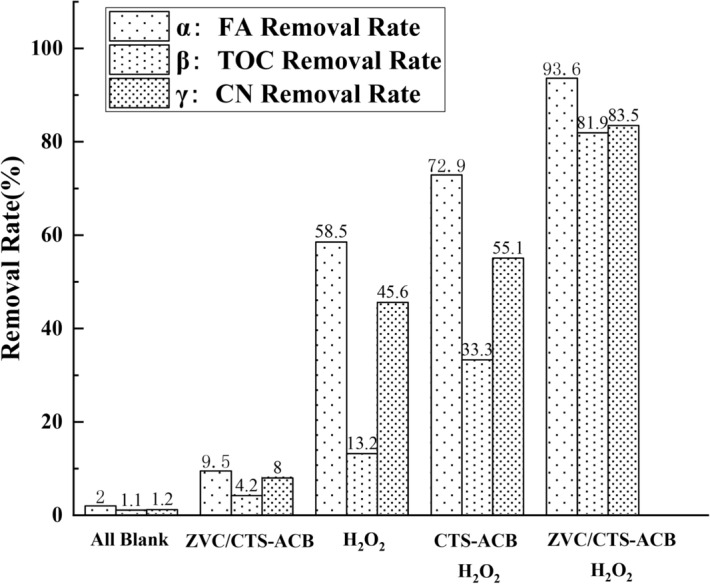
Catalyst and oxidant controlled trials (FA = 100 mg/l, temperature = 94 °C, time = 90 min, pH 3.8, CTS-ACB and ZVC/CTS-ACB = 4 g/l, H_2_O_2_ = 20 mmol).

The removal rates of FA, TOC, and CN expressed by α, β, and γ respectively were shown in Fig. 6. It can be seen from the figure that the addition of oxidant H_2_O_2_ is crucial. The experimental groups with H_2_O_2_ added had better removal rates of FA, TOC and CN compared to those without H_2_O_2_. Among the latter three groups, the groups with catalyst achieved a higher removal rate than the group without catalyst. The removal rates of the three indicators had increased, especially the removal rate of TOC had improved significantly, from 13.2 to 81.9%, which indicates that the addition of ZVC could greatly increase the mineralization rate of FA in the CWPO reaction. Under the same condition, the mineralization rate was nearly 2.5 times higher than that of the experimental group without ZVC. ZVC also has obvious effects on FA degradation and color removal. All the results show that the ZVC/CTS-ACB catalyst prepared in this research has high catalytic activity in FA removal and organic compound mineralization.

The possible degradation mechanism might be: the reaction starts with the Cu^0^ and H_2_O_2_, producing Cu^+^ and OH^-^, Cu^+^ reacts with H_2_O_2_ producing ·OH and Cu^2+^. The generated ·OH could mineralize FA into small molecules, even to CO_2_ and H_2_O.

The stability of ZVC/CTS-ACB was investigated through FA degradation repeating experiments in which the catalyst was repeated five times. The FA removal decreased from 94.78 to 90.05% in five runs. The ICP analysis of the effluent in the first run and fifth run showed that, the leaching concentrations of Cu ion were 0 mg/l and 0.08 mg/l respectively, which demonstrate the recyclability and stability of ZVC/CTS-ACB.

## Conclusion

This study demonstrates the applicability of the prepared ZVC/CTS-ACB to the degradation of FA in CWPO. The statistical tools Plackett–Buiman and central composite design coupled with the response surface model were used to analyze the experimental data and predict the optimal target response value. Under the optimal conditions given by the system, the experiment was repeated three times. The average removal rate of FA was 93.02%, which was very close to the predicted target response value of 93.86%, indicating that the model was established accurately. The experiment results confirm that the PB experiment and the CCD-RSM are suitable for optimizing the operating conditions in a multi-factor operating environment to obtain the maximum FA degradation rate. The comparison experimental results showed that the catalyst and oxidant were essential factors in the CWPO reaction. The ZVC/CTS-ACB catalyst could greatly increase the mineralization rate of FA. The TOC removal rate of 81.9% indicated that most FA were directly mineralized into CO_2_ and H_2_O during the CWPO process, in which ZVC/CTS-ACB showed the high catalytic activity. The low leaching rate of copper ion also showed that the catalyst had good stability. Further study will be conducted on the catalytic mechanism of ZVC/CTS-ACS to FA removal and application of the catalyst in leachate treatment with CWPO.
